# Assessing the Adherence to Antidiabetic Medications Among Patients Diagnosed With Type 2 Diabetes Mellitus in Ajman, UAE

**DOI:** 10.7759/cureus.49325

**Published:** 2023-11-24

**Authors:** Sahil Asgar Ali Shaikh, Jaya Kumari, Yousef Bahmanshiri

**Affiliations:** 1 Internal Medicine, Gulf Medical University, Ajman, ARE; 2 Epidemiology and Biostatistics, Gulf Medical University, Ajman, ARE

**Keywords:** public health, patient compliance, anti-diabetic medications, non-adherence, type 2 diabetes

## Abstract

Background

Medication adherence plays a vital role in managing blood sugar levels and preventing complications in individuals with diabetes. Patient adherence to antidiabetic medications and the factors associated with medication adherence were assessed.

Objectives

To assess the medication adherence among patients suffering from type 2 diabetes mellitus. To determine the various factors influencing medication adherence.

Methods

This cross-sectional study was conducted on patients with type 2 diabetes who were visiting the Internal Medicine Department of Thumbay University Hospital in the United Arab Emirates. A questionnaire was used to gather information about the medication adherence of a group of chosen consecutive patients. IBM SPSS Statistics for Windows, Version 27 (Released 2020; IBM Corp., Armonk, New York) was used for data analysis. A two-sided P-value <0.05 was regarded as significant when using the chi-square test to investigate the relationships between categorical variables.

Results

A total of 204 patients participated in the study: 112 (54.90%) males and 92 (45.09%) females. The mean age of the patients was 49 years. The adherence rates among males and females were 91% and 90%, respectively. Some of the common reasons for non-adherence to antidiabetic medications in our study included forgetfulness, unpleasant side effects, the use of multiple drugs, and long treatment duration.

Conclusion

Our study highlighted important factors associated with patients' non-adherence to their antidiabetic medications. Future research on methods to increase adherence rates should be taken into consideration.

## Introduction

Persistent hyperglycemia is a hallmark of diabetes mellitus (DM), a chronic metabolic disease. It may be caused by decreased insulin secretion, resistance to insulin's effects, or both [1]. According to the International Diabetes Federation report from 2021, 537 million adults aged 20-79 years are currently living with diabetes. This number is projected to increase to 643 million by 2030 and 783 million by 2045 [2]. In the UAE, recent studies have shown that the prevalence of type 2 diabetes was around 25.1% among UAE nationals and 19.1% among expatriates [3].

Healthcare providers need to tailor treatment regimens effectively in diabetic patients with comorbidities to maximize the general well-being of the patient and reduce the possibility of side effects or potential interactions [4]. In individuals with diabetes mellitus, chronic hyperglycemia can exacerbate other metabolic abnormalities and harm several organ systems, potentially resulting in fatal and debilitating health issues such as diabetic retinopathy, diabetic nephropathy, and diabetic neuropathy [5].

Patients with diabetes mellitus need to be on antidiabetic drug therapy as it plays a vital role in glycemic control [6]. Therefore, adherence to medication is highly important. Forgetfulness, interference of medication with lifestyle, and a lack of family and social support are all linked to medication non-adherence [7,8]. In recent times, the use of mobile health apps, which offer functionalities like glucose monitoring, medication reminders, lifestyle tracking, etc., have shown improved adherence rates among diabetic patients [9]. The integration of artificial intelligence and machine learning in wearable devices holds the potential to revolutionize proactive and personalized care among patients living with type 2 diabetes [10].

Medication adherence refers to taking the right dose at the right time as prescribed by the healthcare provider [11]. WHO research states that in developed countries, the average percentage of long-term drug adherence for chronic diseases is about 50%, and that this number is even lower in developing countries. The survey showed that, despite the availability of modern and efficient treatment options, the range of adherence for medications is 31%-71% and substantially lower for lifestyle recommendations [12].

As diabetes is on the rise and adherence to antidiabetic medications remains an ongoing problem, this study was undertaken with the aim of assessing the adherence to antidiabetic medication among diabetic patients and the factors associated with it.

## Materials and methods

A cross-sectional research design was adopted to determine adherence to antidiabetic medications among patients with type 2 diabetes mellitus visiting the Internal Medicine Department of Thumbay University Hospital from November 2022 to February 2023. Patient responses were recorded using a standardized questionnaire administered by the researcher, featuring both open-ended and closed-ended questions.

The estimated sample size was calculated using the formula n = z²p·q/d², where n is the estimated sample size; z the standard value for a 5% level of significance (z = 1.96); d, the margin of desired error, taken as 5%; p the prevalence rate of diabetes = 0.181, and q = 1 - p [2]. The calculated estimated sample size was 228 patients. A systematic random sampling technique was employed to select patients until the estimated sample size was reached. Twenty-four patients refused to participate in the study, so the total number of participants was 204.

The questionnaire was created by the authors, referring to Morisky’s Medication Adherence Scale [13]. Subject matter experts who validated the questionnaire included Internal Medicine department specialists. Content and face validity of the questionnaire were established by reviewing the relevance and appropriateness of each question. A pilot study was conducted on 20 participants, after which the questionnaire was modified to make the questions short and clear for patients.

The questionnaire had four domains: sociodemographic characteristics (age, gender, nationality, education, occupation, marital status); details of diabetes mellitus (age of onset, duration, family history); factors related to medication adherence (patient-related, healthcare provider-related, treatment-related factors); and medication history.

Inclusion criteria comprised all type 2 diabetes patients taking antidiabetic drugs, regardless of nationality. The study excluded patients who expressed a lack of interest.

Self-reported medication adherence to antidiabetic medications by the patients was used to measure adherence rates, as it is feasible in terms of both time and cost.

Before the study began, approval was acquired from the Thumbay University Hospital ethics committee. Written consent was obtained from participants prior to administering the questionnaire.

The data collected were analyzed using IBM SPSS Statistics for Windows, Version 27 (Released 2020; IBM Corp., Armonk, New York). Descriptive and analytical statistics were used to express results. The Chi-square test was used to ascertain the association between medication adherence and associated factors. Statistical significance was set at a p-value less than 0.05.

**Figure 1:**
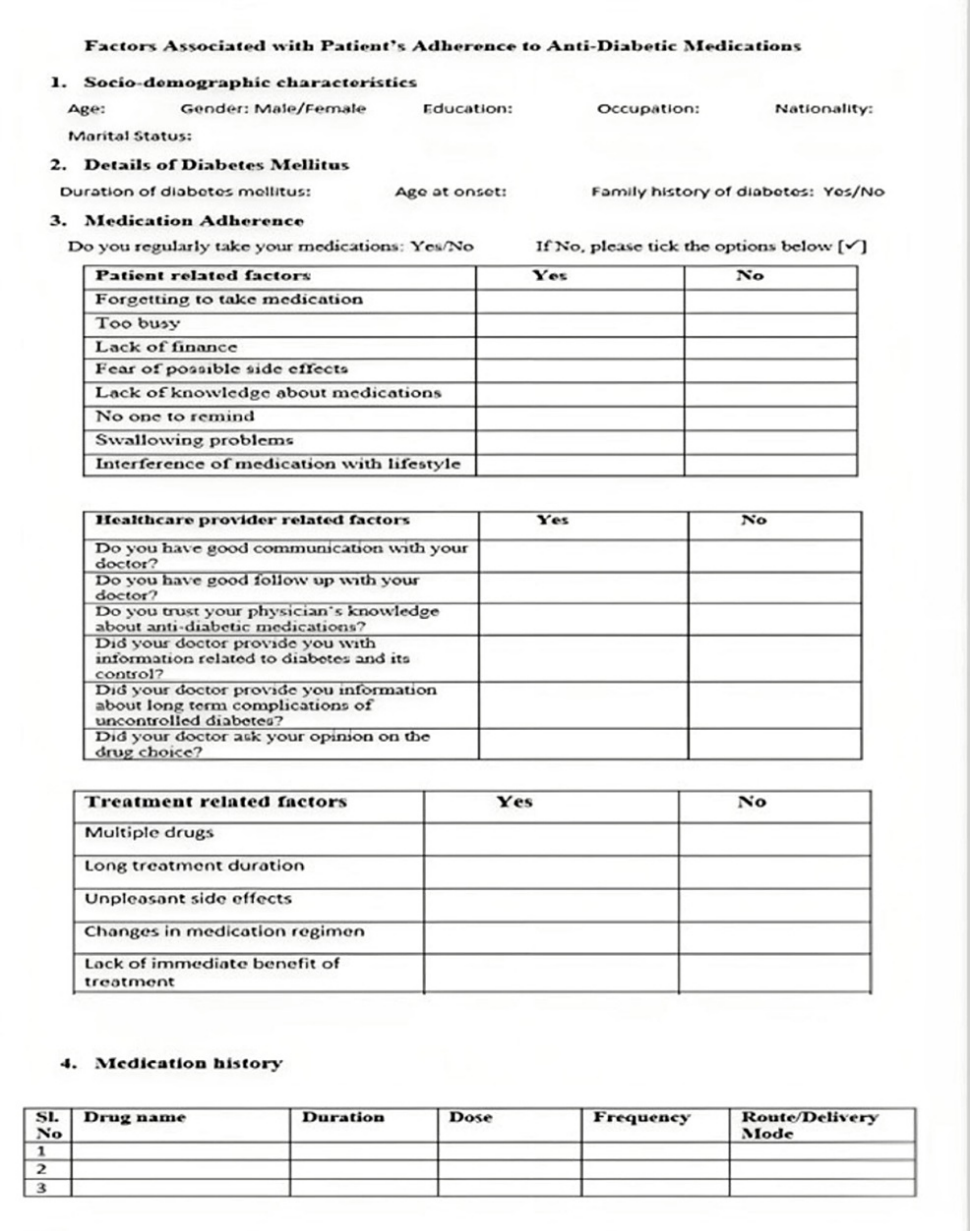
Questionnaire on factors associated with patient’s adherence to antidiabetic medications

## Results

A total of 204 patients were interviewed; 112 (54.90%) males and 92 (45.09%) females. The mean age of the participants was 49 years (SD 10.7), with a minimum age of 28 years and a maximum of 80 years. Regarding the patients' education profiles, 41 (20%) participants had a bachelor’s degree, 18 (8.82%) had a master’s degree, 107 (52.45%) had completed their secondary education, and 38 (18.62%) were illiterate. A total of 171 (83.82%) participants were married, and 33 (16.17%) were unmarried, widowed, or divorced. A total of 38 (18.62%) participants were younger than 40 years of age, whereas 166 (81.37%) participants were 40 years of age or older.

A total of 185 (90.68%) patients self-reported adherence to their antidiabetic medications. The proportion of male participants adhering to their antidiabetic medications was found to be around 91%, whereas the adherence rates among female participants were around 90.21%. However, this difference was not statistically significant.

**Figure 2:**
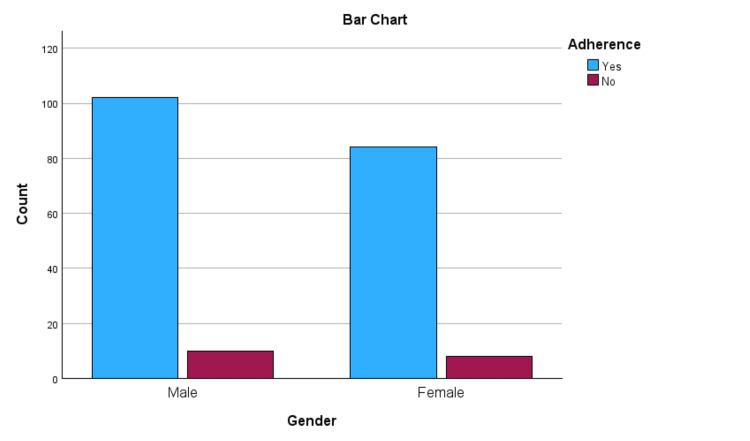
Gender versus adherence.

Participants' adherence to their antidiabetic medications was found to be higher among those who had education up to secondary school (96.26%). Patients with bachelor's and master's degrees had adherence rates of 87.80% and 88.88%, respectively. Illiterate participants reported an adherence rate of 78.94%, but these findings were not statistically significant. It was noted that adherence rates among the married population were 93.56%, whereas the adherence rates among the unmarried population were 75.7%. This finding was found to be statistically significant (p=0.038) (Table 1).

**Table 1:** Sociodemographic characteristics of patients and their adherence to antidiabetic medications.

Sociodemographic factors	Groups	Adherence		Total	p-value
		Yes (%)	No (%)		
Gender	Male	102(91.0)	10(8.92)	112	0.953
	Female	83(90.21)	9(9.78)	92	
Education	Illiterate	30(78.94)	8(21.0)	38	0.110
	12^th ^grade	103(96.26)	4(3.73)	107	
	Bachelor	36(87.80)	5(12.19)	41	
	Masters	16(88.88)	2(11.1)	18	
Age	<40	32(84.21)	6(15.78)	38	0.636
	40+	153(92.16)	13(7.83)	166	
Marital status	Married	160(93.56)	11(6.43)	171	0.038
	Unmarried/widowed/divorced	25(75.7)	8(24.2)	33	
Duration of diabetes	<5 years	78(86.66)	12(13.33)	90	0.125
	5-10 years	76(93.82)	5(6.17)	81	
	>10 years	31(93.93)	2(6.06)	33	

Out of the 185 patients who self-reported adherence, 95 (51.35%) were Indians, 46 (24.86%) were Pakistanis, 26 (12.74%) were Bangladeshis, and only 18 (9.72%) were Arabs. Among the 19 non-adherent patients, 5 (26.31%) were Indians, 7 (36.84%) were Pakistanis, 4 (21.10%) were Bangladeshis, and 2 (10.5%) were Arabs.

Approximately, 73.6% of the patients were non-adherent due to forgetting to take their medication, and around 68.4% of patients reported non-adherence due to fear of side effects. Other factors contributing to non-adherence included patients being too busy, lack of knowledge about medications, and interference of medications with lifestyle (Table 2).

**Table 2:** Self-reported reasons for non-adherence to antidiabetic medications.

Patient-related factors	No. (n=19)	Percentage (%)
Forgetting to take medication	14	73.68
Too busy	10	52.63
Lack of finance	4	21.0
Fear of possible side effects	13	68.42
Lack of knowledge about medications	8	42.10
No one to remind	6	31.57
Swallowing problems	3	15.7
Interference of medication with lifestyle	5	26.31

A good number of patients, 174 (94.05%), reported that they had trust in their physician’s knowledge about antidiabetic medications. Approximately 98.91% of the patients reported that their physician provided them with information related to diabetes and its control. Also, 91.89% of the patients reported that they could communicate well with their physician. Around 73.51% of the patients were also informed about the long-term complications of diabetes by their physician (Table 3).

**Table 3:** Healthcare provider-related factors contributing to non-adherence to antidiabetic medications.

Healthcare provider-related factors	Adherent group, n=185		Non-adherent group, n=19	
	Yes (%)	No (%)	Yes (%)	No (%)
Good communication between physician and patient	170(91.89)	15(8.10)	11(57.89)	8(42.10)
Good patient follow-up by the doctor	108(58.37)	77(41.62)	6(31.57)	13(68.42)
Trust in physician’s knowledge about anti-diabetic medications	174(94.05)	11(5.94)	15(78.94)	4(21.05)
Your physician provided information on diabetes and its control	183(98.91)	2(1.08)	7(36.84)	12(63.15)
Your physician provided information on long-term complications of uncontrolled diabetes	136(73.51)	49(26.48)	7(36.84)	12(63.15)
The physician asked your opinion on the drug choice	97(52.43)	88(47.56)	9(47.36)	10(52.63)

Approximately, 78.94% of the non-adherent patients also reported having unpleasant side effects due to their medications. Use of multiple drugs, changes in medication regimen, and long treatment duration were some other factors contributing to non-adherence (Table 4).

**Table 4:** Treatment-related factors contributing to non-adherence to antidiabetic medications.

Treatment-related factors	Adherent group, n=185		Non-adherent group, n=19	
	Yes (%)	No (%)	Yes (%)	No (%)
Multiple drugs	84(45.40)	101(54.59)	14(73.68)	5(26.31)
Long treatment duration	142(76.75)	43(23.24)	13(68.42)	6(31.57)
Unpleasant side effects	71(38.37)	114(61.62)	15(78.94)	4(21.05)
Changes in medication regimen	95(51.35)	90(48.64)	14(73.68)	5(26.31)
Lack of immediate benefit	36(19.45)	149(80.54)	4(21.05)	15(78.94)

It was observed that the majority of the patients (83.24%) in the adherent group were prescribed oral antidiabetic medications by their doctor, which, when compared to the non-adherent group, only 42.10% of the patients were prescribed oral antidiabetic medications. It was found that 57.89% of the patients in the non-adherent group were prescribed insulin, whereas only 8.15% of patients were prescribed insulin in the adherent group. Additionally, 8.64% of the patients in the adherent group were prescribed both antidiabetic medications and insulin. It was also noted that the most common oral antidiabetic medications prescribed to the patients included biguanides, sulfonylureas, gliptins, and alpha-glucosidase inhibitors (Table 5).

**Table 5:** Usage of oral antidiabetic medications and insulin among adherent and non-adherent diabetic patients.

Medications	Adherent group, n=185	Non-adherent group, n=19
Oral antidiabetic medications	154(83.24)	11(57.89)
Insulin	15(8.15)	8(42.10)
Both	16(8.64)	0

## Discussion

In the current study, the adherence rate among males and females was 91% and 90%, respectively. In comparison, a cross-sectional study conducted by Hyder et al. showed that the adherence rates among males and females were 31.2% and 68.8%, respectively [14]. The reasons for high adherence rates among both males and females could be due to effective patient education about diabetes by their doctor, which was observed in 98.91% of the patients. Good patient follow-up with their doctor and effective communication between the doctor and the patient are other factors contributing to high adherence rates among both genders [15].

Regarding educational attainment, the highest adherence rates were observed among patients who had education up to secondary school. Additionally, patients with Bachelor’s and Master’s degrees had good adherence rates of 87.80% and 88.88%, respectively. This, when compared to a study conducted by Siraj et al., found higher adherence rates among patients with Bachelor’s and Master’s degrees [16]. Adherence rates could vary among patients with different levels of education. One reason for increased adherence among patients with a lower educational background could be limited access to other medical alternatives, making them adhere to the treatment regimens provided by their doctor and thereby contributing to high adherence rates.

The UAE consists of a diverse and mixed population encompassing various cultural backgrounds, traditions, and beliefs. In our study, the highest adherence rates were found among Indians, and the highest non-adherence rates were found among Pakistanis. Certain cultural and religious beliefs towards chronic illnesses could potentially play a role in these findings [17].

It was seen that 93.56% of the patients in our study were married, and they reported having better adherence rates compared to the unmarried population. In a study conducted by Wu et al., similar findings were reported. The reason for this finding could be due to increased social support among the married population. Spouses could potentially play an active role in medication management, contributing to better adherence rates [18].

Some common reasons for non-adherence to medications in our study included forgetfulness, unpleasant side effects, use of multiple drugs, and long treatment duration. In comparison, a cross-sectional study conducted by Badi et al. among Sudanese patients showed that fear of side effects was the most common reason for non-adherence to medication [19].

Approximately 52.63% of patients in our study reported they were too busy, which led to non-adherence to their medication. This contrasts with the cross-sectional study conducted by Leopold et al. among African patients, where only 11.3% of patients reported being too busy [20].

It was also observed that 94.05% of patients had trust in their physician’s knowledge about antidiabetic medications. This is similar to the results of a cross-sectional study conducted by Lucine et al. among Lebanese patients [21]. Having trust in the physician contributes to a sense of emotional support from their physician and allows shared decision-making, resulting in better adherence rates.

Forgetfulness to take the medication could be due to several reasons, such as the use of multiple drugs, which often involves taking multiple doses at different times of the day, making it easy to miss one or more doses. Other reasons for forgetfulness include having a busy lifestyle or changes in the daily routine. Unpleasant side effects of the medication are another reason contributing to non-adherence, as they can affect the patient’s quality of life, causing them to skip doses. Long treatment duration is another factor causing non-adherence, as it can be demotivating for patients to take medications for an extended duration [15].

However, several strategies can be followed by patients to overcome their adherence challenges. These can include the use of medical reminder apps, pill organizers, or alarms to help remind them to take their medication on time. Having open communication with the doctor is also very important, as this can help the doctor identify the patient's concerns and make appropriate adjustments to their treatment plans. Also, providing patients with a better understanding of the treatment regimen and its long-term benefits can keep them motivated, which can lead to better adherence rates [22].

The subjectivity of the data on medication adherence, which may be influenced by recall bias, is one of this study's drawbacks. Another limitation is that it is a questionnaire-based study, which has its own set of limitations, such as response bias by the participants. However, because self-reported surveys are feasible in terms of cost and time commitment, they are commonly utilized in research. Another drawback of the study is the lack of generalizability. The self-reported medication adherence to the medications was not confirmed with blood glucose levels in this study.

## Conclusions

In conclusion, the medication adherence rates among males and females in our study were found to be 91% and 90%, respectively. Some of the common reasons for non-adherence to medications included forgetfulness, unpleasant side effects, use of multiple drugs, and long treatment duration. To improve adherence rates, strategies such as the use of pill organizers, medical reminder apps, and alarms could be employed. Additionally, resolving worries and misunderstandings, offering constant support, and encouraging a cooperative healthcare environment might motivate patients to take an active role in managing their diabetes through regular medication adherence.
